# Application of the implementation research logic model for evaluating a framework for disseminating arthritis-appropriate evidence-based interventions

**DOI:** 10.3389/frhs.2025.1630135

**Published:** 2025-08-06

**Authors:** K. L. Carluzzo, L. E. Bernstein, J. Chevan, E. Erck, H. Murphy, T. Radske-Suchan, B. Engebretsen, K. E. Schifferdecker

**Affiliations:** ^1^ Center for Program Design and Evaluation, The Dartmouth Institute for Health Policy and Clinical Practice, Geisel School of Medicine, Dartmouth College, Lebanon, NH, United States; ^2^ Center for Advancing Healthy Communities, National Association of Chronic Disease Directors, Decatur, GA, United States; ^3^ Iowa Community HUB, Des Moines, IA, United States; ^4^ Primary Health Care, Des Moines, IA, United States; ^5^ The Dartmouth Institute for Health Policy and Clinical Practice, Geisel School of Medicine, Dartmouth College, Lebanon, NH, United States

**Keywords:** arthritis, arthritis-appropriate evidence-based intervention, community care hub, evaluation, implementation, implementation research logic model, physical activity, referral

## Abstract

The Public Health Framework for Collaborative Arthritis Management and Wellbeing (“the Framework”) is being piloted as a model to improve health and wellbeing of people with arthritis. This model is built upon a foundational partnership between a clinical entity and a community care hub (“hub”), which recognizes the important role that hubs play in addressing both chronic care needs and unmet social needs of people with arthritis. Specifically, hubs partner with healthcare systems by coordinating and supporting networks of community-based organizations that provide patients with access to health-related resources and services [(such as Arthritis-Appropriate Evidence-Based Interventions (AAEBIs)]. In this Framework, the clinic engages patients in screening (based on physical activity, physical function, and pain), counseling (regarding benefits of physical activity), and referral to community care hubs. At the hub, patients are screened for unmet social needs and are matched with AAEBIs based on a shared-decision-making process. There are two types of AAEBIs: Physical Activity, and Self-Management Education, which may be offered in community-based, clinical, or virtual settings. Through the screening, counseling, and hub referral process, the pilot Framework seeks to increase identification of people who would benefit from AAEBIs, increase AAEBI participation among those who would benefit, and ultimately improve the health and wellbeing of people with arthritis. The evaluation of this Framework leverages an Implementation Research Logic Model (IRLM) and its component frameworks and taxonomies in methods and outcome selection. This study follows the implementation of the Framework through key stages: screening, brief advice and counseling, referral to hub, AAEBI selection and participation, outcomes measurement, and feedback of data to the clinic. This paper offers a practical example of the iterative process we used to make decisions for the evaluation, how the IRLM is used to guide decision-making and analysis, and the methods of our evaluation plan.

## 1 Introduction

Arthritis is the main cause of disability in the United States (1) with 21.2% of Americans reporting an arthritis diagnosis (2). Osteoarthritis (OA), which is the most common form of arthritis, impacts about 33 million adults in the United States (3). Physical activity is an effective treatment option for OA, as it is associated with improvements in pain, energy, sleep, and physical function (4).

In an effort to increase physical activity and self-management for OA, the United States' Centers for Disease Control and Prevention (CDC) and the Osteoarthritis Action Alliance (OAAA) recommend Arthritis Appropriate Evidence-Based Interventions (AAEBIs). Programs, which are run by non-profit Community-Based Organizations (CBOs), must demonstrate statistically significant improvements in at least two arthritis-relevant outcomes (e.g., pain, balance, physical function, disability) to be an endorsed AAEBI (5). AAEBIs such as the Arthritis Foundation's Walk with Ease or Better Choices Better Health meet these requirements and have been shown to improve patients' self-efficacy and balance in addition to reducing disability, pain, and fatigue (6, 7).

Despite these benefits, AAEBIs are underutilized by patients with arthritis (8). In recognition of this lack of uptake, the CDC focused on providing additional funding to promote their use, including the State Public Health Approaches to Addressing Arthritis (CDC-RFA-DP-23-0001) (9). One aspect of these efforts is to include clinicians such as primary care providers to help increase AAEBI engagement, as patients with arthritis are more likely to engage in physical activity when it is recommended to them by a physician (10, 11). However, given high workload and competing priorities, many primary care providers and rheumatologists are not recommending physical activity for their patients with OA (12, 13).

To facilitate patient referrals to AAEBIs while managing provider burden, the National Association of Chronic Disease Directors (NACDD), in collaboration with the CDC, developed the Public Health Framework for Collaborative Arthritis Management and Wellbeing. NACDD is a professional, non-profit association of healthcare professionals that aim to promote health and reduce the burden of chronic disease. This Framework identifies community care hubs (“hubs”), as a mechanism for facilitating referrals. Hubs utilize established community partnerships to connect patients to CBO-offered health programs (e.g., AAEBIs), which alleviates the administrative burden on providers in the AAEBI selection and referral process (14).

The Framework was developed with input from an expert panel involving rheumatology, primary care, and chronic disease management professionals. It was designed to meet the following goals: (1) improve identification of patients with OA who would benefit from AAEBI participation, and (2) connect those patients to AAEBIs to improve their physical activity and health-related quality of life (HRQOL) while utilizing a workflow that is feasible to implement in clinical and community-based settings. In the application of this Framework, the hub plays a central role in ensuring continuity of care for patients with arthritis by connecting the healthcare delivery system with a number of Community-Based Organizations that offer AAEBIs.

We developed an Implementation Research Logic Model (IRLM) specific to the Framework to structure and convey the multiple theoretical frameworks and taxonomies used to evaluate relevant aspects of this pilot implementation. The IRLM is a tool that was developed from an NIH-identified need to improve the rigor, reproducibility, and transparency of implementation projects (15, 16). The IRLM captures the multi-level nature of the Framework and cross-organization involvement to promote AAEBI participation.

Evaluation of this Framework will assess (1) whether patients experience improvement in HRQOL, (2) the feasibility and acceptability of implementing this Framework in a primary care setting and a community hub, and (3) the return on investment of the Framework implementation. This evaluation focuses on both the pilot site implementation and its implications for broader Framework application. In this paper, we describe the process of developing an evaluation plan for the Framework, including the role of IRLM and component models (e.g., Consolidated Framework for Implementation Research) and resulting decisions that were made.

## 2 Methods

**Figure 1:**
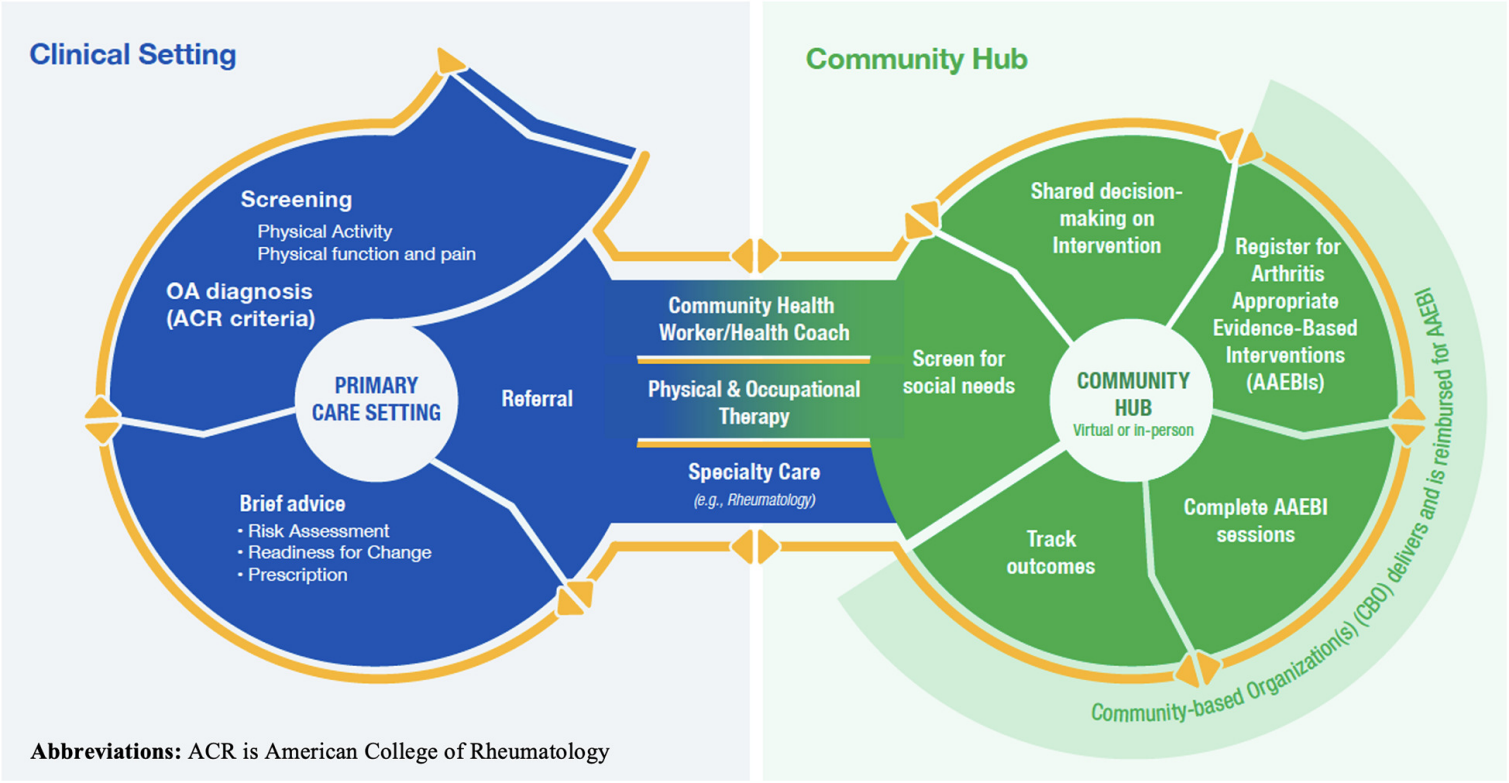
The public health framework for collaborative arthritis management wellbeing. This image is used with the permission of the National Association of Chronic Disease Directors.

Figure 1 illustrates the Framework and shows the multiple steps involved in the process of identifying and referring eligible patients with OA to AAEBIs. In the clinical setting, this includes screening patients with a pre-existing hip or knee OA diagnosis for current levels of physical activity, providing brief advice and counseling on the benefits of physical activity, and referring patients to a hub. On the hub side, navigators screen patients for health-related social needs, engage in a shared decision-making process with patients to identify the available AAEBI that is most appropriate for them, facilitate their enrollment in AAEBIs, and track AAEBI completion. Sufficient technology is required to facilitate many aspects of this process, notably at the interface between healthcare providers and community hub, to facilitate the sending of referrals and return of outcomes data.

### 2.1 Theoretical approach for the evaluation

The Implementation Research Logic Model (IRLM) draws on several leading frameworks and taxonomies to guide the evaluation (16). Determinants (evidence-based factors that influence implementation outcomes) include barriers or facilitators to implementation. Our IRLM drew on the most recent version of the CFIR determinant framework (17, 18) to specify determinants within each domain: intervention characteristics, inner setting, outer setting, characteristics of individuals, and process. Implementation strategies, which are influenced by determinants, are system-level changes or supports needed to support implementation. These are aligned with the Expert Recommendations for Implementing Change (ERIC) (19). Implementation mechanisms (by which the strategies impact the outcomes) are denoted with the three components of the behavior change model known as COM-B: Capability (C), Opportunity (O), Motivation (M), along with Behavior (B) (20). The outcomes we selected are organized by the types of outcomes described in Proctor's taxonomy of outcomes for implementation research (21): implementation, service, and client (described here as “recipient”).

**Figure 2:**
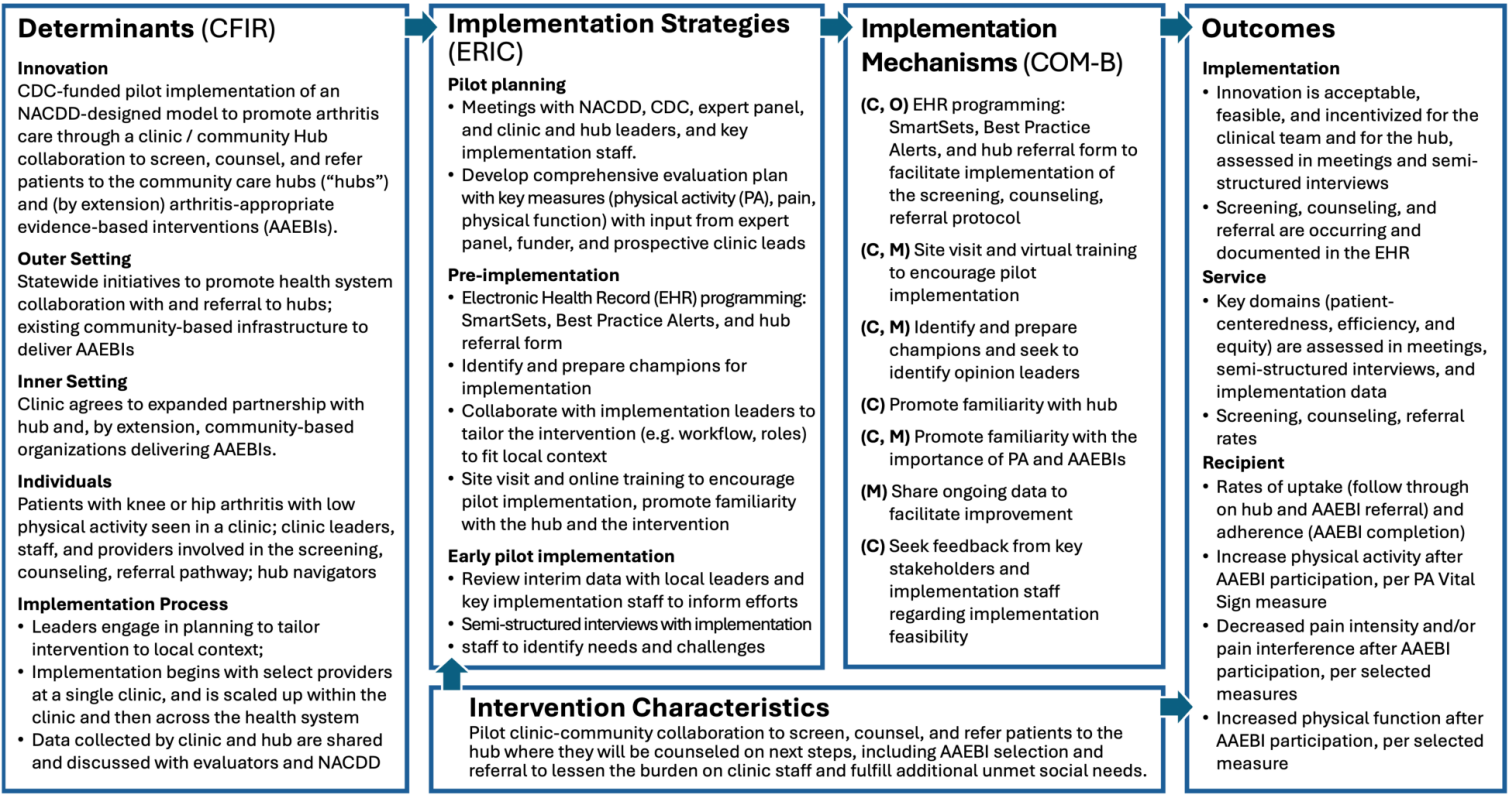
Implementation research logic model for the arthritis management framework.

During the process of developing the Framework, our evaluation team partnered with NACDD and CDC to iteratively develop the evaluation plan that aligned with the components described in Figure 2. Key phases of this process included: (1) a focused literature review, to identify similar studies and the evaluation frameworks, measures, and methods used; (2) Understanding partner priorities for the evaluation through meetings bi-weekly with NACDD, monthly with CDC, and periodically with the expert panel; and (3) Drafting and revising an evaluation plan and its components with multiple rounds of feedback from NACDD, CDC, the expert panel, and (eventually) the pilot site.

Guided by the IRLM and the five specific aims of the project, we developed a detailed Evaluation Plan and Data Collection Protocol by Study Aim (Table 1). The overall study design is a longitudinal transformation mixed methods design (22, 23). In this approach, qualitative and quantitative data are collected over time, and data are analyzed and triangulated throughout to further explain findings (e.g., showing quantitative data about patients screened to clinicians during interviews to further understand facilitators and barriers to screening) and to identify potential additional topics to explore. As detailed in Table 1, we will collect quantitative data for formative evaluation and quality improvement (monthly) and for outcomes assessment (1–2 times annually) using multiple data sources, including the clinic's electronic health record (EHR), referrals, AAEBI participation, patient surveys of key outcome measures, billing code usage, and staff surveys. We will use qualitative data primarily for formative evaluation with data derived from document review (e.g., meeting notes), and semi-structured interviews.

**Table 1:** Evaluation plan and data collection protocol by study aim.

Implementation aims	Source/Method	Measure	Analysis
Patient level			
1. Improve physical activity and health-related quality of life of adults with knee or hip arthritis	Electronic Health Record (EHR) data	Proportion of eligible patients screened, counseled, referred per EHR data	QI run charts
	Hub data	Proportion of referred patients who enroll/complete AAEBI	Summary comparison
	EHR and/or hub data	Collection of Physical Activity Vital Sign (PAVS), and Patient Reported Outcome Measure Information System (PROMIS (Physical Function, Pain Interference, Pain Intensity) Individual-level change in PAVS and PROMIS measures	Baseline and post referral assessments
2. Patients who are screened, counseled, referred to hub, and participate in Arthritis-Appropriate Evidence-Based Interventions (AAEBIs) are representative of the practice's eligible population	EHR data	Proportion of eligible patients in selected demographic characteristics	Summary comparison
	Hub data	Proportion of referred patients in selected demographic characteristics	Summary comparison
	Patient interview	CFIR domains (selected)	Thematic analysis
Providers/Practice level			
3. Implementing the Framework is acceptable, feasible, and incentivized for the clinical team	Patient interviews	CFIR domains (selected)	Thematic analysis
	Clinical team interviews	CFIR domains (selected)	Thematic analysis
	Clinical team	CFIR domains (selected)	Survey (brief)
	Meeting notes	CFIR domains (selected)	Review
4. Referral to and implementation of AAEBIs is acceptable and feasible for the hub	Hub leader or referral lead interviews	CFIR domains (selected)	Thematic analysis
	Meeting note review	CFIR domains (selected)	Thematic analysis
	Hub referral data	Proportion of pilot practice referrals received	Summary comparison
System level			
5. Implementing screening, counseling, and AAEBI referrals provides revenue return on investment for practice and cost savings	Billing data	Proportion of qualifying visits that use intervention-related billing codes (e.g., G2211)	Summary analysis
	EHR data	Population-level change in falls risk per STEADI	Baseline and post referral assessments

AAEBI, arthritis-appropriate evidence-based intervention; CFIR, consolidated framework for implementation research; EHR, electronic health record; PAVS, physical activity vital sign; PROMIS, patient-reported outcomes measurement information system; PT, physical therapy; QI, quality improvement.

Throughout the evaluation planning period, we were mindful to reduce the potential implementation burden placed on patients, primary care practice staff, and the hub team. A working group that is comprised of the evaluation team, primary care practice and hub leadership, and members of the NACDD team iteratively sought feedback from practice and hub staff along with an expert advisory panel. This feedback was used to select screening and outcome measure of reasonable lengths, adjust the timing of screening, counseling, and referral to fit within existing workflows, and shorten and revise provider educational modules about the Framework.

Below we describe specific considerations in the development of our evaluation plan. Dartmouth College's Committee for the Protection of Human Subjects deemed the evaluation as not human subjects research (#00032976).

### 2.2 Measure selection

A key consideration in measure selection was to identify instruments that could easily be implemented in the practice and hub settings or were already used in those settings, and, as applicable, could serve dual purposes of screening and baseline and outcome measurement. We consulted with primary care practice staff at the pilot site, and they provided feedback about the permissions needed to integrate measures into their electronic health record (EHR), the impact of measure length on provider time capacity, the value of measures for clinical decision making, and readily available data that could be used as measures. The hub also provided feedback on which measures were already being collected by their team as part of AAEBI-specific screening and follow-up requirements. This feedback, along with considerations about the reliability and validity of each measure and concern for patient and provider burden, was used to select the final measures.

Through this process, the domains of physical activity, physical function, and pain (interference and/or intensity) were identified as screening and outcome measures. The 2-item Physical Activity Vital Sign (PAVS; initially termed “Exercise Vital Sign” (24); was selected to assess physical activity. Scores on the PAVs are associated with health outcomes such as BMI in arthritis patients and in other clinical settings (25). The Patient Reported Measurement Information System® (PROMIS) Short Form v2.0 – Physical Function (PF) 4a was proposed as a brief assessment of physical function but was replaced by the PF-10a due to presence in EHR and existing clinical implementation. The PROMIS Short Form v1.1 – Pain Interference 4a and single-item Pain Intensity scale (0–10) were selected to assess pain. The latter was replaced by another 0–10 visual analog scale (Baker Wong FACES) due to existing presence in EHR and clinical implementation. The PROMIS Pain Interference and Physical Function scales, like other PROMIS measures, were developed through a rigorous process that included testing and calibration (26) and have been validated in osteoarthritis samples (27). The Baker Wong FACES scale, which was originally developed to measure pain in pediatric patients (28), has been validated in adult samples (29) and has shown sensitivity in measuring chronic osteoarthritis pain (30). Additional process measures (e.g., number of patients screened or referred) and outcome measures related to practice revenue (use of billing codes specific to screening for physical activity and counseling and ongoing care, e.g., Medicare code G2211) and reducing potential health system costs were also identified. Given that fall rates are associated with increased health system costs (31), reduction in falls risk will be a proxy measure of health system savings and will be measured by the CDC's Stopping Elderly Accidents, Deaths, and Injuries (STEADI) scale (32). The STEADI, which measures gait, strength, and balance, has been shown to independently predict fall risk (33).

### 2.3 Defining eligible participants and sampling

As with other aspects of the evaluation plan, the process for determining eligibility for the screening-counseling-referral intervention was iterative and informed by feasibility and appropriateness at the clinic site. For example, initially proposed eligibility criteria for screening were more expansive, and included all adult patients who presented with hip or knee pain or who had been diagnosed with hip or knee osteoarthritis. However, to first trial the pilot on a smaller scale, participation was limited to patients who have: a qualifying visit type (chronic care management, Medicare Annual Wellness Visit, or Medicare Welcome Visit), a diagnosis of osteoarthritis of the knee or hip, and a knee or hip-related complaint. There are plans to continue implementation beyond the pilot and expand patient eligibility criteria to additional visit types and those with hip and knee pain without an OA diagnosis. These decisions will be informed by the results of this evaluation.

**Figure 3:**
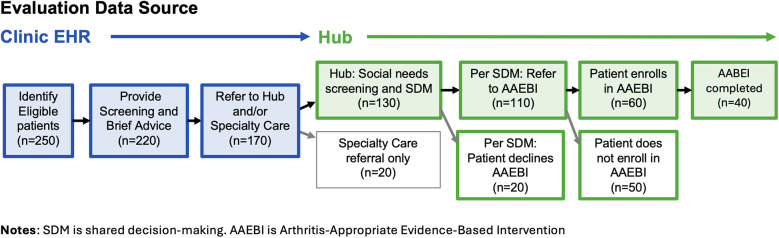
Participant flow and estimated retention.

The flow of eligible patients and estimated numbers for this study are specified in Figure 3. Some attrition is expected at each stage, the reasons for which will be assessed in provider and staff interviews and hub data. Interviews with providers will investigate the reasons for which not all eligible patients are screened or provided with brief advice regarding physical activity, and insights regarding the decision to refer to the hub, specialty care, or neither. Interviews with hub staff and documentation in hub database will shed lights on reasons some patients, as a result of the shared decision-making process, decline to participate in AAEBIs, and reasons that some patients choose not to enroll once they are referred. As implementation progresses, patient criteria may be expanded to include additional clinics, visit types, and those who have not yet been diagnosed with hip or knee arthritis.

For the qualitative data collection, we plan to conduct interviews with patients that have completed a hub referral and AAEBI participation and interviews and surveys with clinic and hub staff. Interviews will aim to understand patient and provider experiences engaging in screening, counseling, and referral including the value for patients and barriers and facilitators to implementation and engagement. Interviews with key implementation staff and providers will be conducted approximately three months from the start of implementation to assess experiences with initial implementation to inform potential modifications to implementation. If there are delays in implementation, additional interviews will be conducted earlier to assess barriers and needs. We will use purposive sampling with patients to ensure a mix of ages, race/ethnicity, and socio-economic status and will include all clinic and hub staff involved in the flow of care for eligible patients or are in a leadership position that oversees aspects of the pilot implementation.

### 2.4 Data sharing and management

To determine the necessary level (patient, population), type (specific measures), frequency (monthly, semi-annually), and source (clinic, hub) of data needed to fulfill the evaluation plan, the relevant parties involved in overseeing and facilitating the extraction and receipt of data (evaluators, health system and hub representatives) met to discuss their respective interests, needs, and limitations. Sample data extracts were reviewed collaboratively and data extraction requirements, specifications, and quality assurance processes were agreed upon.

Prior to the current implementation effort, a standing Business Associate Agreement existed between the clinic and the hub, which allowed for data related to patient referrals to be shared from the clinic to the hub and data related to patient participation in AAEBIs and outcomes to be shared from the hub to the clinic. A data sharing agreement was established between the hub and the evaluation team at Dartmouth to allow for deidentified patient-level data to be shared for the purposes of quality improvement and evaluation.

To facilitate data matching across the clinic and hub, the referral ID will be used to match clinic and hub records. Clinic staff responsible for preparing data to be shared with the evaluation team will follow internal quality assurance procedures to ensure that the data are provided based on specifications, including matching with hub data. Quality control checks will be performed by clinic staff and evaluators on the deidentified data to ensure it meets specifications and to resolve inconsistencies or issues that may be identified. Timely reporting is contingent upon timely data processing and provision.

### 2.5 Analysis and reporting

As with other aspects of the study, decisions regarding the frequency and contents of reporting were made collaboratively between the evaluation and implementation teams. Decisions were informed by feasibility (e.g., frequency of manual data extraction, and matching), appropriateness (e.g., based on eligible patient volume).

#### 2.5.1 Quantitative analysis

We will first assess pilot implementation by analyzing the proportion of eligible patients that complete the full process of screening, counseling and referral. Conversations with the primary care and hub teams revealed that access these data will inform ongoing implementation efforts, indicating the need for potential modifications. Therefore, the decision was made to report these numbers to the clinical team using monthly run charts to aid in quality improvement.

In addition to monthly run charts, we will analyze and report on patient-level program outcomes. Specifically, we will assess whether patients show changes in physical activity, physical function, pain, and falls risk after completing an AAEBI. We plan to conduct separate paired samples t-tests for these four outcomes, comparing patients' baseline scores with data collected after AAEBI completion. For these analyses, we will use casewise deletion to exclude any patients that do not have both baseline and post-program data. We conducted an *a priori* power analysis using G*Power (34) to calculate the minimum sample size needed to detect a medium effect size (0.5), as per Cohen's recommendations (35). To achieve 80% power with a significance level of less than 0.05, a sample size of at least 34 patients is required. However, if this minimum is not met or the data at both time points is not normally distributed, we will conduct the non-parametric Wilcoxon signed-rank test to assess baseline to post-program change. We will also calculate the program completion rates of those referred to either AAEBIs or physical therapy.

Subgroup descriptive analyses will also be conducted to understand how the demographic breakdown of patients that are referred to the hub align with the demographics of those that are eligible. We plan to analyze subgroups based on demographic variables such as age, gender, and race. However, final decisions will depend on patient demographic distribution and data volume.

The primary care team provided estimates of the number of patients expected to enroll in the program (Figure 3). To ensure adequate data volume to conduct the proposed analyses, the decision was made to extract data at six and twelve months after the start of implementation. An interim summary of results will be shared with the implementation team at six months with more expansive reporting happening after a year of implementation. We also consulted with the hub team to understand the type and data they are already reporting for other initiatives to align the timing and structure of the data to reduce potential burden.

#### 2.5.2 Qualitative analysis

Qualitative analysis of semi-structured interviews and bi-weekly implementation team meeting notes will be used to evaluate implementation acceptability and feasibility. As noted previously, will be conducted approximately three months from the start of implementation to assess experiences with initial implementation to inform potential modifications to implementation. If there are delays in implementation, additional interviews will be conducted earlier to assess barriers, needs, and relevant contextual factors.

Methodologists from the evaluation team will analyze meeting notes and transcripts using web-based qualitative analysis software (e.g., Dedoose). These data will be analyzed using a mixed inductive (data-driven) and deductive (theory-driven) approach, guided by the current CFIR, including a codebook template created by the CFIR Research Team-Center for Clinical Management Research, which covers the various domains and subdomains of the current CFIR.

Inter-coder dependability will be established and maintained using several processes. First, we will create clear code definitions and refine as needed through review and consensus of at least two coders, including tracking decisions and reasons for changes. Second, two coders will each code the first three to four documents of each data type and compare coding to establish reliability and agreement on codes, code applications, and code definitions. Finally, once consensus is reached on the codebook, one coder then will do the majority of the coding, with coding checks on approximately 20% of the coded data and continued discussion and refinement for any questions or coding discrepancies. For identification of themes, we will use code applications and code co-occurrences with analytic memos in Dedoose to explore patterns and arrive at the preliminary themes. These will be reviewed by the full study team to consolidate the findings and finalize the themes. Saturation will be determined when no major new codes or themes emerge as we progress through the analysis.

#### 2.5.3 Triangulation

We will use two of the four major types of triangulation described by Denzin (36): methodological triangulation and investigator triangulation. We will use methodological triangulation by comparing results across different methods (e.g., screening and referral data, interviews, meeting notes) and investigator triangulation by always having at least two evaluators involved in data collection and analyses and involving other research team members in review and discussion of findings across our methods. Results of preliminary analyses and monthly tracking will be used to inform staff interviews. The CFIR will be used to guide the assessment of domains between qualitative and quantitative findings to identify relevant results. The results of the triangulation, and reflection upon these results with the implementation team, will help inform decisions related to modifying and scaling the pilot.

## 3 Discussion

By drawing on key domains within the IRLM, such as feasibility, acceptability, engagement, and adaptability of both the intervention and the evaluation components, we were able to effectively engage the relevant partners involved in implementation to tailor the evaluation plan to the pilot. This process clarified which elements of the initial evaluation plan were essential, alterable, or non-essential. The interactive online version of the IRLM was particularly helpful in facilitating application to our project – a draft of which we then refined through further reflection on the literature and on the specific models encompassed therein.

Measure alignment across research, evaluation, and clinical care is a perennial challenge. As a pilot evaluation, we sought to be flexible in the development of the evaluation. This meant identifying the measures and timing of data collection along with the eligible visit types that were most feasible and least burdensome to both patients and those involved in the implementation, while still creating a standardized evaluation plan with rigorous measures, processes, and requirements needed to assess key outcomes. To the extent possible, we leveraged measures that were already being collected across entities or could serve multiple purposes (e.g., patient screening and outcomes assessment).

While limiting patient eligibility to Medical Annual Wellness visits and those with official hip and knee OA diagnoses increases the real-world feasibility of the pilot implementation, this may compromise the ability to generalize findings to other patients. Additionally, while we plan to analyze subgroup differences in patients' outcomes and engagement, we will be limited by the sample size of the pilot, limiting conclusions about the Framework's impact across demographic groups. However, once the initial feasibility of the Framework implementation is established, we anticipate expanding the eligible visit type, which will likely increase the sample size of enrolled patients, and thus improve inclusivity.

Not all implementations of the Framework will look the same. The specific partners that engage in future implementations, with their unique strengths, limitations, infrastructure, and interests, will influence site readiness, the feasibility of implementation and decisions made along the way. However, use of the IRLM as we illustrate draws attention to the CFIR determinant framework to assess new contexts and readiness for implementation, including the inner setting, outer setting, characteristics of individuals, and existing processes. Based on our experience, factors such as readiness for change, eligible patient volume, payer mix, technology to support referrals, having a local hub, and local Community Based Organizations offering AAEBIs are all relevant and will need to be assessed and supported to guide additional local implementations.

The conclusion of the pilot and evaluation will call for reflection on the Public Health Framework for Collaborative Arthritis Management Wellbeing as it was initially conceived and consider, in light of the IRLM and evaluation results, what aspects are fixed or adaptable to allow for adoption in all relevant areas of care. Providing guidance on fixed and flexible aspects of the Framework may encourage adoption and fidelity. For example, the Framework may be applied in other clinical settings (e.g., physical therapy), or in other community-based settings (e.g., direct partnership with AAEBI providers). As such, responsibility for core components of the Framework (e.g., shared decision-making for AAEBI selection) may need to shift.

This work contributes to models where there is a partnership between clinical and community systems and collaboration is required to acknowledge the different needs, interest, capabilities, incentives of each partner.

## Data Availability

The original contributions presented in the study are included in the article/Supplementary Material, further inquiries can be directed to the corresponding author.
